# Cortico-muscular coherence in primary lateral sclerosis reveals abnormal cortical engagement during motor function beyond primary motor areas

**DOI:** 10.1093/cercor/bhad152

**Published:** 2023-05-04

**Authors:** Saroj Bista, Amina Coffey, Antonio Fasano, Teresa Buxo, Matthew Mitchell, Eileen Rose Giglia, Stefan Dukic, Mark Heverin, Muthuraman Muthuraman, Richard G Carson, Madeleine Lowery, Orla Hardiman, Lara McManus, Bahman Nasseroleslami

**Affiliations:** Academic Unit of Neurology, Trinity Biomedical Science Institute, Trinity College Dublin, Dublin 2, Ireland; Academic Unit of Neurology, Trinity Biomedical Science Institute, Trinity College Dublin, Dublin 2, Ireland; Academic Unit of Neurology, Trinity Biomedical Science Institute, Trinity College Dublin, Dublin 2, Ireland; Academic Unit of Neurology, Trinity Biomedical Science Institute, Trinity College Dublin, Dublin 2, Ireland; Academic Unit of Neurology, Trinity Biomedical Science Institute, Trinity College Dublin, Dublin 2, Ireland; Academic Unit of Neurology, Trinity Biomedical Science Institute, Trinity College Dublin, Dublin 2, Ireland; Academic Unit of Neurology, Trinity Biomedical Science Institute, Trinity College Dublin, Dublin 2, Ireland; Department of Neurology, University Medical Centre Utrecht Brain Centre, Utrecht University, Utrecht 3584 CG, The Netherlands; Academic Unit of Neurology, Trinity Biomedical Science Institute, Trinity College Dublin, Dublin 2, Ireland; Neural Engineering with Signal Analytics and Artificial Intelligence, Department of Neurology, University Hospital Würzburg, Würzburg 97080, Germany; Trinity College Institute of Neuroscience and School of Psychology, Trinity College Dublin,, Dublin 2, Ireland; School of Psychology, Queen’s University Belfast, Belfast BT71NN, UK; School of Electrical and Electronic Engineering, University College Dublin, Dublin 4, Ireland; Academic Unit of Neurology, Trinity Biomedical Science Institute, Trinity College Dublin, Dublin 2, Ireland; Beaumont Hospital, Dublin 9, Ireland; Academic Unit of Neurology, Trinity Biomedical Science Institute, Trinity College Dublin, Dublin 2, Ireland; Academic Unit of Neurology, Trinity Biomedical Science Institute, Trinity College Dublin, Dublin 2, Ireland

**Keywords:** primary lateral sclerosis (PLS), cortico-muscular coherence, upper motor neuron, neurodegeneration EEG, EMG

## Abstract

Primary lateral sclerosis (PLS) is a slowly progressing disorder, which is characterized primarily by the degeneration of upper motor neurons (UMNs) in the primary motor area (M1). It is not yet clear how the function of sensorimotor networks beyond M1 are affected by PLS. The aim of this study was to use cortico-muscular coherence (CMC) to characterize the oscillatory drives between cortical regions and muscles during a motor task in PLS and to examine the relationship between CMC and the level of clinical impairment. We recorded EEG and EMG from hand muscles in 16 participants with PLS and 18 controls during a pincer-grip task. In PLS, higher CMC was observed over contralateral-M1 (α- and γ-band) and ipsilateral-M1 (β-band) compared with controls. Significant correlations between clinically assessed UMN scores and CMC measures showed that higher clinical impairment was associated with lower CMC over contralateral-M1/frontal areas, higher CMC over parietal area, and both higher and lower CMC (in different bands) over ipsilateral-M1. The results suggest an atypical engagement of both contralateral and ipsilateral M1 during motor activity in PLS, indicating the presence of pathogenic and/or adaptive/compensatory alterations in neural activity. The findings demonstrate the potential of CMC for identifying dysfunction within the sensorimotor networks in PLS.

## Introduction

Primary lateral sclerosis (PLS) is a slowly progressive disorder of upper motor neuron (UMN) degeneration (Finegan et al. 2019). A definite diagnosis of PLS requires clinical signs of UMN dysfunction, a disease duration of at least 3 years, and an absence of the significant lower motor neuron (LMN) degeneration that differentiates it from amyotrophic lateral sclerosis (ALS) (Turner et al. 2020). PLS is also characterized by cortical and subcortical changes beyond primary motor area (M1) and the corticospinal tracts (Finegan et al. 2019). These widespread structural changes in the sensorimotor network are likely to, in turn, impact the function and neural communication between different parts of this network. Such potential differences in the interactions between different parts of the sensorimotor network during motor tasks can be best assessed by quantifying the fast oscillatory interactions between neuroelectric signal recordings (Coffey et al. 2020; Dukic et al. 2021).

Recent electroencephalogram (EEG) studies have demonstrated a correspondence between neuroelectric activity and UMN pathology in ALS (McMackin et al. 2019). The high temporal resolution of EEG is well suited to provide information concerning rhythmic or oscillatory brain activity across a range of frequencies. Previous EEG investigations in people with ALS, conducted at rest, have demonstrated an altered functional connectivity across brain networks in the theta (4–7 Hz) and gamma (31–60 Hz) frequency bands (Westphal et al. 1998; Blain-Moraes et al. 2013; Nasseroleslami et al. 2017; Dukic et al. 2019). The UMN pathology of ALS is similar to that of PLS, though ALS has both UMN and LMN pathology. This suggests that cortico-cortical communication is also similarly altered in PLS, and this is supported, in part, by magnetic resonance imaging (MRI) studies (Agosta et al. 2014; Meoded et al. 2015).

The functional significance of abnormal cortical communication can be better understood by examining cortical engagement during active motor tasks. More specifically, the cortical regions engaged in the execution of motor tasks can be assessed by calculating the coherence between ensemble neural activity recorded over cortex (measured with EEG) and the collective activity of spinal motor neurons recorded from the contracting muscle (EMG) (Conway et al. 1995; Halliday et al. 1998). Cortico-muscular coherence (CMC) is typically observed as synchrony (in the beta and gamma bands) between EEG electrodes over M1 and EMG activity. It is considered to be indicative of the efferent drive to the spinal motoneurons while also being subject to the modulating influence of peripheral afference (Witham et al. 2011). Peak beta-band CMC over M1 is reduced in conditions characterized by UMN degeneration, including stroke (Fang et al. 2009; Aikio et al. 2021) and ALS (Issa et al. 2017; Proudfoot et al. 2018). In principle, CMC also provides a method of investigating whether changes in the engagement of other cortical regions during movement accompany the PLS-induced neuronal loss in M1. It might be anticipated that the loss of fast-conducting corticospinal axons in PLS will be accompanied by pathogenic, adaptive, and/or compensatory changes throughout the sensorimotor network, given the redundancy in the sensorimotor system (Neilson and Neilson 2005; Ajemian et al. 2013; MacKinnon 2018). This may involve the engagement of cortical regions beyond M1 (Bede et al. 2021).

Here, we used EEG and EMG signals recorded during the performance of a motor task to test the hypothesis that, in PLS, CMC can be detected over brain regions extending beyond M1. We also sought to determine whether the variations in CMC are correlated with the clinical measures of UMN dysfunction.

## Materials and methods

### Ethics

The study was approved by the “Tallaght University Hospital/St. James’s Hospital Joint Research Ethics Committee—Dublin,” REC Reference: 2019-05 List 17 (01), and was performed in accordance with the Declaration of Helsinki. All participants provided informed written consent to the procedures before undergoing assessment.

### PLS cohort

The PLS cohort were prospectively recruited in this cross-sectional study between June 2017 and August 2019 through the national ALS clinic at Beaumont Hospital. All participants with PLS fulfilled the clinical criteria for PLS (Turner et al. 2020). Healthy controls, age-matched to the PLS cohort, were recruited from a database of healthy controls interested in taking part in the ongoing research studies in the Academic Unit of Neurology, Trinity College Dublin, the University of Dublin.

Subjects with a history of major head trauma or other neurological conditions that could affect cognition, alcohol dependence syndrome, current use of neuroleptic medications, or high-dose psychoactive medication were excluded. Those with diabetes mellitus, a history of cerebrovascular disease, and those with neuropathy from other causes were also excluded. All of the PLS cohort underwent nerve conduction studies and EMG to exclude other concurrent peripheral nerve disorders that could interfere with CMC analyses.

### Clinical assessment

On the day of EEG recording, the PLS cohort underwent an extensive clinical assessment. Disease duration from the symptom onset and the site of disease onset were recorded. Muscle strength was assessed using the Medical Research Council (“MRC”) score (Compston 2010) in 9 bilateral (i.e. 18) upper limb muscles, including deltoid, triceps, biceps, wrist flexors and extensors, fingers flexors and extensors, and abductors of the index fingers and thumbs. The degree of clinical UMN involvement in the upper limbs was graded by a UMN score (de Carvalho et al. 2003). An adapted UMN score based on Kent-Braun et al. (1998) was calculated using reflex and UMN signs assessment. Reflexes were assessed at 3 sites in the upper limbs (biceps, triceps, and brachioradialis). The UMN score ranges from 0 (normal) to 16 (reflecting hyperreflexia [0–6], hypertonia [0–4], clonus [0–2], Babinski [0–2], and Hoffmann sign [0–2]). The Edinburgh Cognitive ALS Screen (ECAS), which evaluates cognitive performance across language, verbal fluency, executive, memory, and visuospatial domains (Abrahams et al. 2014), was performed on 14 of the 16 PLS participants (2 declined). Edinburgh handedness inventory (Oldfield 1971) with 10 questionnaires was performed to assess the handedness of the participant.

HD-EEG and bipolar surface EMG were subsequently recorded in all participants for the calculation of CMC during motor tasks.

### Experimental paradigm

**Fig. 1:**
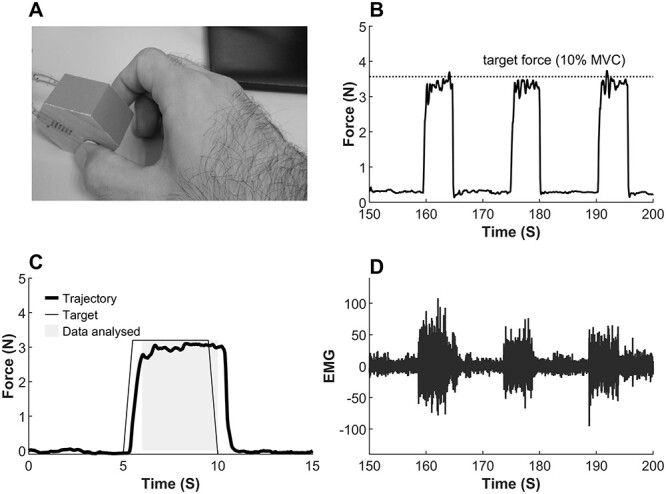
A) Pincher grip motor task performed by thumb and index finger of right hand. B) A segment of force profile of pincher grip motor task performed at 10% of maximal voluntary contraction (MVC). C) A force trajectory of the pincher grip motor task averaged over 30 trials. D) Segment of EMG signal recorded from the FDI muscle during 10% MVC pincher grip motor task.

Assessment was conducted in the same manner for the PLS and control groups, similar to previous work carried out by our group, and as described in detail by Coffey et al. (2020), with additional notes in Supplementary files. Participants held a force transducer between the thumb and the index finger of their right hand to measure the pincer grip force (Fig. 1A). The maximal voluntary contraction (MVC) was determined as the average peak force achieved during 3 short (5 s) maximal contractions, where the peak force in these attempts lay within 10% of each other. Similar to our previous study, participants were asked to produce a force at 10% MVC for 5 s, while holding the force transducer in pincer grip, guided by visual force feedback on screen (pincer grip task). In a second task, participants were also asked to hold the force transducer for 5 s (precision grip task). Preliminary analysis showed that participants exhibited lower beta-band CMC during the pincer grip task compared with precision grip (Supplementary Fig. S1). The present study focused on the pincer grip task, as the preliminary CMC analysis at the sensor level indicated greater differences between PLS and controls during this task. Participants attempted a total of 30 trials for each task.

### Recording of (neuro-)electro-physiological signals

All participants were seated comfortably, and the EEG data were recorded in a special-purpose laboratory, using 128-channel scalp electrode cap, filtered over the range of 0–400 Hz and digitized at 2,048 Hz using the BioSemi ActiveTwo system (BioSemi B.V, Amsterdam, Netherlands). Each participant was fitted with an appropriately sized EEG cap.

Surface EMG data were recorded using a bipolar electrode configuration from 8 muscles in the right upper arm, with the electrode pairs placed in accordance with the SENIAM guidelines (Hermens et al. 2000). The online hardware gain and filter settings for the EMG signals during recordings were the same as EEG channels, which was followed by further offline preprocessing. Five EEG channels (Cz, Pz, C4, Fz, and C3) and 3 EMG signals (first dorsal interosseous [FDI], flexor pollicis brevis [FPB], and abductor pollicis brevis [APB]) were chosen a priori for the CMC analysis. The EEG electrodes were chosen due to their representative coverage of the cortical motor network. The C3, Cz, and C4 cover the contralateral hand area, central, and ipsilateral hand sensorimotor regions for the chosen tasks. Fz pertains to the frontal areas that reflect the activity from supplementary motor regions (and, to some extent, premotor areas). Finally, Pz reflects the activity from parietal areas that play important roles in visuomotor tasks (Nasseroleslami et al. 2014). Importantly, these regions have minimal spatial overlaps and allow the activity of more distinct regions to be assessed. The target muscles were selected based on their biomechanical involvement in the pincer grip task (Danna-Dos Santos et al. 2010).

### Signal preprocessing and spectral analysis

**Fig. 2:**
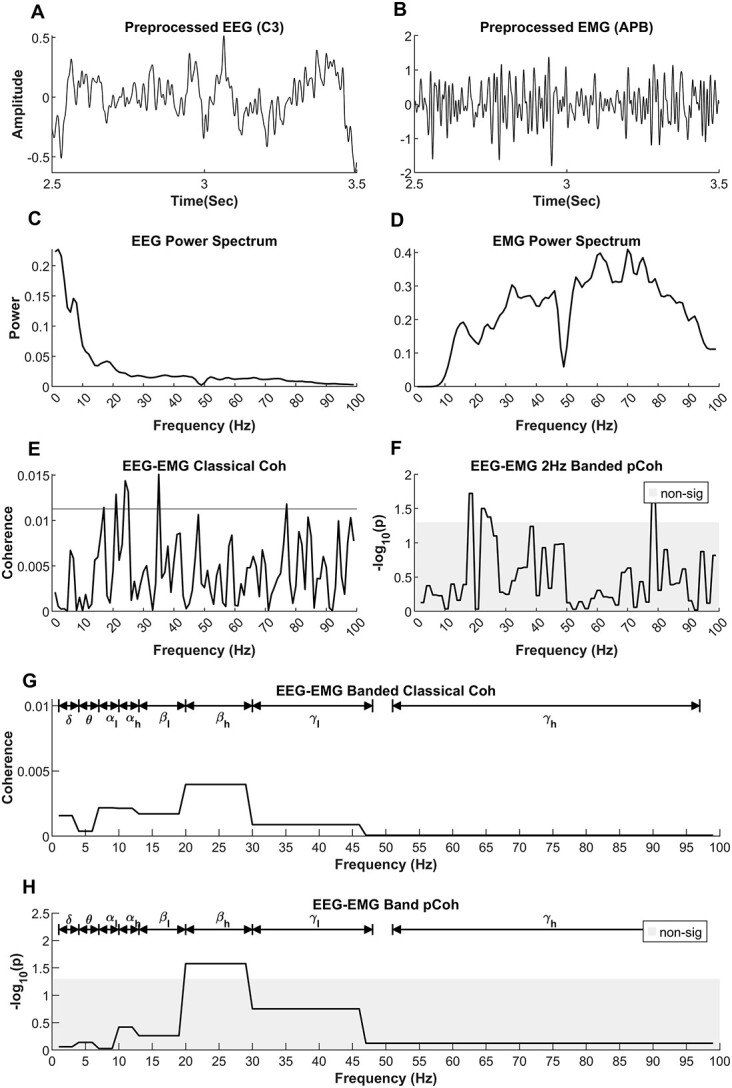
Example showing the estimation of CMC using data from a healthy control participant (CON13 in Supplementary Fig. S2). A) Preprocessed EEG signal recorded from C3 electrode. B) Preprocessed EMG signal recorded from APB muscle during the same time. C) Power spectrum of EEG signal. D) Power spectrum of EMG signal in the frequency range of interest. E) CMC estimated using the magnitude squared coherence with spectral smoothing (“classical coherence”). F) CMC calculated using the spatial median to estimate the auto- and cross-spectra of the EEG and EMG data (“pCoh”). Here, the spatial median was used to group the coherence spectra over bands with a 2-Hz interval to facilitate the comparison of pCoh with classical coherence. G) Conversion of classical magnitude squared coherence into banded CMC values. Here, the spatial median method is used to group the classical coherence spectra so that there is 1 coherence value for each of the predefined bands. H) pCoh CMC calculated using the spatial median method to group coherence spectra over predefined bands. Note that images (F) and (H) use the same coherence methodology, with the only difference being the bandwidth of the frequency bands used for grouping the coherence spectra. Frequency bands: delta (δ), theta (θ), low alpha (α_l_), high alpha (α_h_), low beta (β_l_), high beta (β_h_), low gamma (γ_l_), and high gamma (γ_h_).

EEG/EMG data analysis (Fig. 2) was performed as described in detail in a previous study (Coffey et al. 2020). Briefly, automated artifact rejection routines (Fieldtrip Toolbox) (Oostenveld et al. 2011) were used to discard data contaminated by noise. After visual inspection of the 128-ch recordings, EEG channels with higher levels of noise were removed and were reconstructed using weighted average interpolation of neighboring channels (Perrin et al. 1989). An average of 22 ± 6 trials (i.e. 88 ± 24 s) for the 5 target EEG channels were retained for the corticomuscular coherence calculation across all participants. A time window/epoch duration of 4 s (starting 1 s after the visual cue) was chosen for analysis. Data epochs, where the coefficient of variation of the force produced was >0.2, or where the mean force was <8% or >20% MVC, were excluded from further analysis. An average of 3 ± 6 trials (i.e. 12 ± 24 s) data were removed across all participants for these reasons. The raw EEG data were (re-)referenced using surface Laplacian spatial filter (McFarland et al. 1997; Bradshaw and Wikswo 2001), which served to provide signals that are more spatially specific to each EEG electrode. The EMG data (signal amplitude) were normalized with respect to root mean square EMG amplitude at 100% MVC. EEG and EMG data were filtered between 1–100 and 10–100 Hz, respectively, using a dual-pass fourth-order Butterworth bandpass filter. The auto-spectrum of each EEG/EMG signal, and the cross-spectrum between all combinations of EEG–EMG signals (frequency resolution: 1 Hz, bandwidth: 2–100 Hz) were calculated using Fieldtrip toolbox (Hanning taper and frequency smoothing at 1 Hz, nonoverlapping windows of 1 s). EMG signals were not rectified.

### Estimation of coherence spectrum and banded coherence

Coherence is presented based on equivalent z-scores and *P*-values at both subject and group levels. This approach prevents bias by eliminating the dependence on the number of trials for the coherence analysis.

CMC was examined in 8 different frequency bands, and a single coherence estimate was obtained for each band—delta (2–4 Hz), theta (5–7 Hz), low alpha (8–10 Hz), high alpha (11–13 Hz), low beta (14–20 Hz), high beta (21–30 Hz), low gamma (31–47 Hz), and high gamma (53–97 Hz, excluding the 48–52 Hz range to avoid mains power noise). The frequency bands were defined based on the typical physiological EEG frequency bands (Sanei and Chambers 2007) as well as their relevance both in sensorimotor control (Nasseroleslami et al. 2014) and quantifying network dysfunction in motor neuron diseases (Dukic et al. 2019, 2021).

CMC was estimated based on the spatial median using the following procedure. Coherence was estimated using the median value of the auto- and cross-spectra represented by their real and imaginary components in the 2D space calculated across epochs (Weiszfeld 1937; Niinimaa and Oja 2014) and Figure 2 in Nasseroleslami et al. (2019). This contrasts with classical coherence estimates which are based on the expected value or arithmetic mean of the spectra. The auto- and cross-spectra for each 1-s epoch were calculated for each participant. The spatial median coherence was then estimated from the spatial median of the auto- and cross-spectra with a resolution of 2 Hz, Fig. 2F, and across each of the 8 defined frequency bands to obtain the “banded coherence,” Fig. 2H. The banded spectral CMC was normalized by dividing the band cross-spectrum by the respective band auto-spectra. The strength of coherence was subsequently presented using the equivalent *P*-value as -log(*P*), which we denote as “pCoh.”

To represent the banded CMC as a probability, each coherence value was compared against 0 using a nonparametric 1-sample test for significant coherence (spatial [signed] ranks; Hannu Oja and Randles 2004; Hannu Oja 2010; Nordhausen and Oja 2011). This procedure yielded individual *P*-values for each frequency band for each individual (both PLS and control groups). Stouffer’s method was used to combine individual *P*-values to derive average *P*-value within each group, i.e. in the healthy group and in the PLS group (Stouffer et al. 1949; Westfall 2014). This procedure is similar, but not procedurally equivalent, to the pooled coherence analysis (Amjad et al. 1997). Both methods can be used to combine information from several participants (or trials). The negative logarithm of the *P*-values, i.e. −log_10_(*P*), was used as a measure of CMC strength to visualize CMC. The band-specific coherence values, expressed in −log_10_(*P*), were used to represent the collective coherence over the range of frequencies within each distinct frequency band (Fig. 2H).

For comparison, the magnitude squared coherence, referred to here as “classical coherence,” was also estimated in the frequency range of 2–100 Hz in addition to the banded coherence. Spectral smoothing of the auto- and cross-spectrum was done using a Hanning filter. The significance threshold (upper 95% confidence limit) was calculated as $1-0.05^{1\kern-2pt\big/\kern-2pt_{\left(L-1\right){}^\ast 0.375}}$, where *L* is the number of segments used to calculate coherence and the factor 0.375 is correction for spectral smoothing using Hanning filter (Halliday and Rosenberg 1999).

### Statistics

To find significant group differences between the banded CMC values, the band-specific CMC values (expressed as *P*-values) were converted into *z*-scores by taking inverse of cumulative distribution function of $1-P$. The resulting *z*-scores of CMC values were compared between controls and PLS using a nonparametric 2-sample Kolmogorov–Smirnov test (Massey 1951), which compares the shapes of 2 distributions rather the central tendency (mean and median). In total, 120 comparisons (5 EEG × 3 EMG × 8 frequency bands) were made. Correction for multiple comparisons was performed using the adaptive false discovery rate (FDR) at *q* = 0.05 (Benjamini et al. 2006). The effect size of the CMC differences was calculated using Cohen’s *d*.

Correlation of the CMC measures with clinically assessed UMN scores was calculated for all the predefined frequency bands and the preselected EEG and EMG channels (i.e. 120 CMC measures in total, 5 × 3 EEG–EMG combinations × 8 frequency bands). The association of the CMC measures, expressed in −log_10_(*P*), in the PLS cohort with their corresponding UMN scores was tested using Spearman’s rank correlation coefficient. For this purpose, partial correlations were used to remove the potential effects of age from the inference (range: 46.39–77.43 years). The *P*-values of correlation coefficients were adjusted for multiple comparison (120 comparisons in total, 5 EEG × 3 EMG × 8 frequency bands) using adaptive FDR at *q* = 0.05. A line was fitted to the correlation data to visualize the relationship using the robust linear least-square fitting method. The degrees-of-freedom-adjusted coefficient of determination (Adj *R*^2^) was calculated for the fitted line to measure the goodness of the fit.

## Results

### Clinical profile

Sixteen participants with PLS (7 females and 9 males, age: 62.7 ± 8.7 [mean ± SD]) with PLS were prospectively recruited from the national ALS Clinic based at Beaumont hospital, Dublin. All participants with PLS were diagnosed with definite PLS, fulfilling the consensus criteria (Turner et al. 2020) defined as the absence of LMN degeneration 4 or more years from symptom onset; 18 healthy controls (7 female) were recruited (age: 62.5 ± 8.97 [mean ± SD]). Table 1 shows the detailed profile of the recruited participants.

**Table 1:** Clinical and demographic data of the analyzed PLS and control groups.

	PLS	Controls
Biological sex (female/male)	7/9	7/11
Average age at recording (years)	62.7 ± 8.7	62.5 ± 8.9
EHI (right/left)	14/2	16/2
Disease duration (years)	7.6 ± 6.01	–
UMN score (max 16)	12.8 ± 2.3	
Spasticity score (upper limb) (max 4)	3.5 ± 1.09	
MRC (upper limb) (max 100)	71.6 ± 4.08	
ECAS total abnormal score *n* (%)	4 (28%)	
Language	1 (7%)	
Verbal fluency	2 (14%)	
Memory	2 (14%)	
Visuospatial	1 (7%)	

EHI, Edinburgh Handedness Inventory; UMN, Upper Motor Neuron Score; MRC, Medical Research Council Scale for Muscle Strength; ECAS, Edinburgh Cognitive ALS Screen.

ECAS results were scored as normal or impaired based on education and age (Pinto-Grau et al. 2017). Four participants with PLS (28%) showed evidence of cognitive impairment based on the total ECAS score. The details are listed in Table 1. Abnormal performance in visuospatial domains (7%) were uncommon based on our screening assessment with ECAS.

### Abnormally high CMC in PLS

**Fig. 3:**
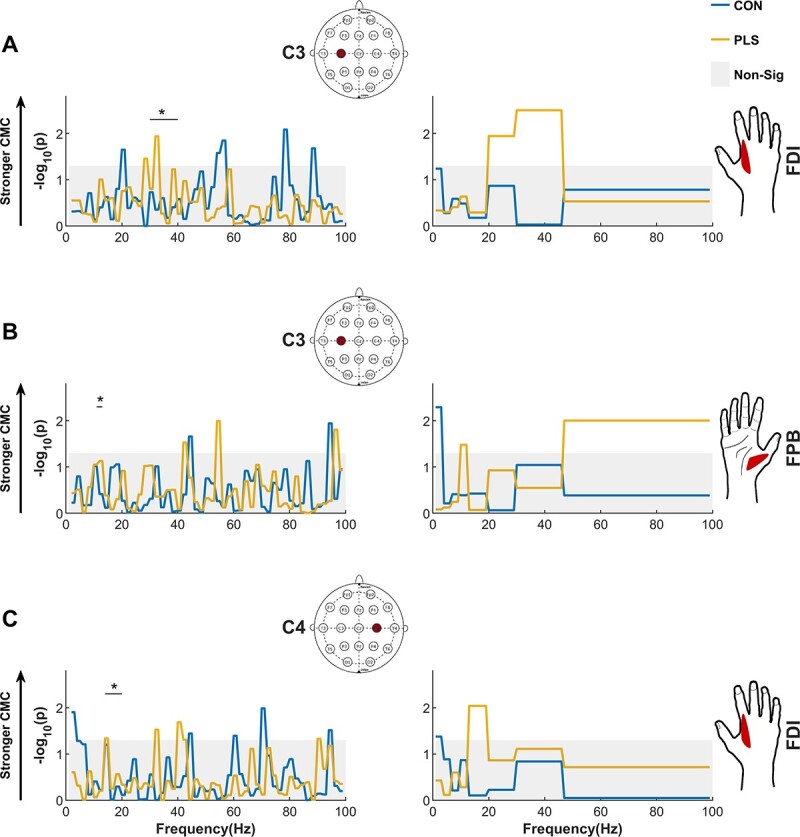
Participants with PLS show abnormal cortico-muscular (EEG–EMG) coherence in primary motor areas and abnormal frequency bands. The first column displays pCoh grouped over shorter 2-Hz frequency bands, and the second column shows the banded coherence (“pCoh”) grouped over predefined frequency bands. The pCoh spectra show the strength of synchrony of the EEG electrodes over the contralateral primary motor area C3 (A and B) and ipsilateral primary motor area C4 (C) with EMG (FDI and FPB muscles) in different frequency bands. The shaded area corresponds to the nonsignificant values at α = 0.05 threshold for *P*-values (corrected for multiple comparison, 120 comparisons in total, using FDR at *q* = 0.05). In PLS, CMC between C3-FDI was present in the gamma band instead of the typical beta-band CMC observed in healthy controls during this type of task. The PLS cohort also exhibited CMC between ipsilateral C4-FDI in the beta band which was not present in controls.

**Fig. 4:**
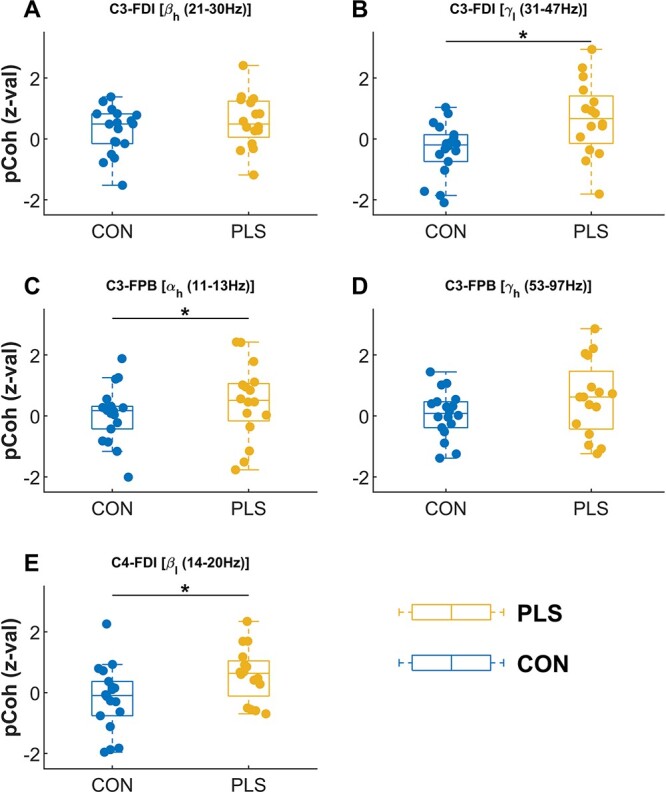
Box plot of banded CMC (expressed as *z*-scores) for the EEG–EMG channel and frequency band combinations that were found to show significant CMC in PLS after FDR correction (based on Table 2, see Fig. 3 and Supplementary Fig. S3). The plots show the CMC, between EEG electrodes over the contralateral primary motor area C3 (A, B, C and D) and ipsilateral primary motor area C4 (E) with EMG (FDI and FPB muscles) in different frequency bands, for control and PLS participants overlayed with individual values. The groups were compared using Kolmogorov–Smirnov test. Significant group difference is marked with an asterisk (^*^P < 0.05, corrected at FDR *q* = 0.05).

The results show that there were statistically significant differences in the frequency, location, and magnitude of the CMC between healthy controls and the PLS group, Fig. 3 (*q* < 0.05, with FDR multiple comparison correction). The coherence spectra for all EEG channels and muscles investigated are presented in Supplementary Fig. S3, and the significant differences between PLS and control groups are summarized in Fig. 4 and Table 2. Healthy controls did not show strong beta-band CMC peaks over the contralateral motor area when grouped across all participants (C3), likely due to the task selection (pincer grip vs. precision grip), Supplementary Fig. S1 and Fig. 3A and B. However, when examined on an individual basis, significant beta-band CMC was detected in 14/18 controls (Supplementary Fig. S2). Biological sex had no effect on the CMC detected in the PLS cohort (*P* > 0.05, tested using Mann–Whitney U test).

#### CMC pattern over contralateral primary motor area

CMC was significantly higher in the gamma and alpha bands in the PLS group when compared with controls. The coherence was not statistically significant for the control group at the C3 channel location over contralateral motor area (between C3 and for both the FDI and FPB muscles, respectively, Fig. 3A and B). It is notable that statistically significant gamma- and alpha-band CMC was observed in the PLS cohort, as this is not typically observed in healthy subjects during low-force muscle contractions.

#### CMC pattern over ipsilateral primary motor area

Significant beta-band CMC (β_l_) was observed between C4 and the FDI over the ipsilateral motor area in the PLS cohort and was not observed in controls (Fig. 3C).

### Correlates with UMN dysfunction score show location-specific positivity and negativity

**Fig. 5:**
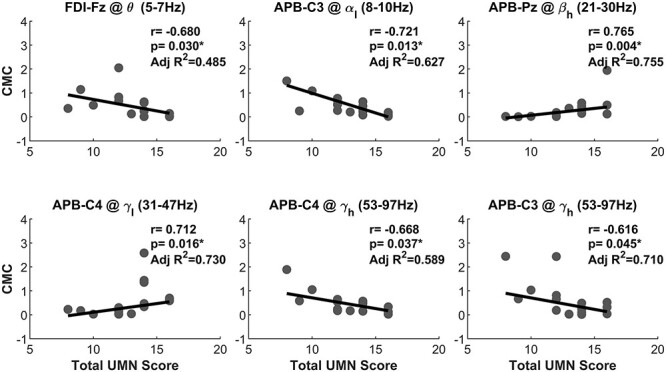
Measures of cortico-muscular (EEG–EMG) coherence in PLS shows significant strong positive and negative associations with the clinically defined UMN dysfunction score (i.e. Spearman’s rho between 0.6 and 0.8 or between −0.8 and −0.6 for each correlation). The *P*-values have been corrected for FDR at *q* = 0.05. Notice that the correlations are partial correlations with the effect of age removed from the inference.

We then conducted a separate analysis to test for significant correlations between CMC and UMN score (calculated for all predefined frequency bands and EEG and EMG channels). Several of the CMC measures were significantly correlated with the UMN dysfunction score after FDR correction (Table 3 and Fig. 5). In Table 3 and Fig. 5, a negative correlation between a CMC measure and UMN score indicates that higher UMN impairment (more severe clinical symptoms) are associated with reduced EEG–EMG synchrony (CMC) in the PLS cohort. A positive correlation indicates that PLS participants with more severe UMN symptoms exhibited stronger CMC in these muscles/brain regions. Both alpha- and gamma-band CMC between the APB muscle and the contralateral motor cortex were lower in PLS participants with more severe UMN impairments (significant negative correlation with UMN score). Theta-band CMC coherence between the FDI and the frontal brain region (Fz) was also significantly lower in PLS participants with greater UMN dysfunction. Gamma-band CMC between the APB and the ipsilateral motor area (C4) varied with the degree of UMN dysfunction. PLS participants with greater UMN impairments exhibited lower CMC in the high gamma band (γ_h_) but higher CMC in the low gamma band (γ_l_). Finally, PLS participants with greater UMN impairments exhibited greater beta-band CMC between the APB and the parietal brain region (beta-band CMC in the parietal region is not typically observed in healthy controls).

**Table 2:** Table showing group average banded Corticomuscular coherence (CMC) values expressed as *P*-values. The CMC measures pertain to selected EEG–EMG channel and frequency band combinations that were significant in the PLS group after FDR correction at *q* = 0.05 (based on Fig. 3 and Supplementary Fig. S3). The CMC values are shown for controls and PLS along with group difference *P*-values and effect size.

EEG/EMG	Frequency	CON Avg pCoh (*P*)	PLS Avg pCoh (*P*)	Kolmogorov–Smirnov test (*P*)	Effect size Cohen’s *d*
C3-FDI	High beta	0.135	0.011	0.465	0.381
C3-FDI	**Low gamma**	**0.093**	**0.003**	**0.006**	**0.987**
C3-FPB	**High alpha**	**0.040**	**0.033**	**0.047**	**0.374**
C3-FPB	High gamma	0.0412	0.009	0.052	0.524
C4-FDI	**Low beta**	**0.788**	**0.009**	**0.015**	**0.786**

The bold values in Table 2 indicate the CMC comparison between controls and PLS marked by an asterisk (*) in Figure 4.

**Table 3:** Summary of cortico-muscular coherence (CMC) measures of interest.

CMC measure	EEG/EMG location	Frequency band	Significant coherence observed in PLS	Significant difference between PLS and controls	Significant +/− correlation with UMN score	
1	C3-FDI	High beta	✓			
2	C3-FDI	Low gamma	✓	✓		
3	C3-FPB	High alpha	✓	✓		
4	C3-FPB	High gamma	✓			
5	C4-FDI	Low beta	✓	✓		
6	Fz-FDI	Delta			−	
7	C3-APB	Low alpha			−	
8	C3-APB	High gamma			−	
9	C4-APB	High gamma			−	
10	C4-APB	Low gamma			+	
11	Pz-APB	Low beta			+	

## Discussion

To date, studies investigating CMC in motor neuron diseases have focused on estimating beta-band CMC between muscles of the hand/arm and M1 as a direct reflection of UMN/LMN pathology (Proudfoot et al. 2018). However, our recent EEG studies in ALS (Dukic et al. 2019; McMackin et al. 2020) and Post-Polio Syndrome (Coffey et al. 2020) suggest that abnormalities in the cortical network activity extend beyond M1 in these conditions, a finding that is also supported by neuroimaging studies (Finegan et al. 2019). We have used CMC to demonstrate how brain activity in participants with PLS differs from that of healthy controls during the performance of a pinch grip motor task. Here, we characterized the engagement of different brain regions by the oscillatory functional coupling between signals recorded from brain and muscle (Fig. 3, and Table 2). In PLS, higher CMC at the contralateral M1 was observed in the gamma and alpha bands when compared with controls. Significant beta-band CMC was also detected in ipsilateral M1, which is not typically observed in healthy participants. In each case, the CMC measures were higher in PLS than in controls, suggesting that these observed differences are unlikely to be attributable to muscle wasting or dysfunction (which would typically decrease CMC). We also identified several CMC measures that were correlated with clinical measures of UMN dysfunction, which were also identified outside of the contralateral primary motor area.

### PLS-specific differences in CMC

Higher alpha- and gamma-band CMC between contralateral M1 and FDI/FPB was observed in PLS when compared to controls, Fig. 4B and C, respectively, with a large difference reported in the gamma band (Cohen’s *d* = 0.987). Altered functional connectivity throughout the sensorimotor cortex has been similarly demonstrated in ALS in resting-state EEG studies (Nasseroleslami et al. 2017). In the present study, gamma-band CMC was detected in participants with PLS during the low-force muscle contractions. This is unusual as the gamma-band CMC is typically only observed in healthy controls during more forceful or dynamic muscle contractions (Omlor et al. 2007; Gwin and Ferris 2012). Previous literature has shown that gamma- and beta-band CMC are present under different conditions and are often inversely related (i.e. when gamma-band CMC increases, beta-band CMC decreases). For example, gamma band coherence appears during strong contractions, with a corresponding reduction in the beta-band CMC and is thought to reflect a stronger excitation of the motor cortex or greater attention to the task (Brown et al. 1998).

The observed broad increase in CMC in PLS may reflect a combination of pathogenic, adaptive, and/or compensatory increases in the synchronization of neuronal groups in response to UMN degeneration and dysfunction in the inhibitory interneuronal circuitry in PLS (Agarwal et al. 2018). Neuronal loss in M1 in PLS and the cortical and subcortical changes beyond M1 are likely to disrupt information flow in both local neural circuits and larger-scale networks. This may require a rebalancing of interregional interactions and a reorganization of the sensorimotor networks that are engaged in processing and transferring information during movement. This, in turn, would manifest as changes to the synchronization patterns across the sensorimotor network and alterations in the coupling between the cortico/subcortical and spinal regions.

Another key finding was the detection of beta-band CMC in the ipsilateral motor cortex in PLS, with a strong difference reported between PLS and controls (Cohen’s *d* = 0.786). Ipsilateral premotor activity has been previously observed in ALS (specifically, in ALS participants who exhibited a greater number of UMN signs relative to LMN symptoms) in an EEG-based investigation of movement-related cortical potentials (Inuggi et al. 2011). It is possible that the increased activation of the ipsilateral sensorimotor cortices is functionally relevant and aids in the performance of the motor task. Ipsilateral cortical activation is increased in other populations in which elements of the cortical network have been damaged, e.g. in stroke, multiple sclerosis, and spinal cord injury (Ward et al. 2003; Lenzi et al. 2007; Prak et al. 2021). Previous studies suggest that ipsilateral M1 aids the contralateral motor cortex in the planning and organization of hand movements (Chen et al. 1997), but it remains unclear whether ipsilateral M1 plays a significant role in mediating the motor command to motoneurons of the hand (Soteropoulos et al. 2011). There is limited evidence to support a monosynaptic pathway to convey direct ipsilateral actions to hand muscles, but it is possible that ipsilateral projections are conveyed through other indirect/polysynaptic pathways (Calvert and Carson 2022). Though data presented in this study cannot elucidate the precise neural circuits and pathways through which ipsilateral M1 signals influence muscle activity, the results demonstrate for the first time that the contributing brain regions in the sensorimotor control are altered in PLS during a motor task. This manifests as a reshaping of synchronous oscillations between cortex and muscle.

### Associations between CMC and clinical scores

**Fig. 6:**
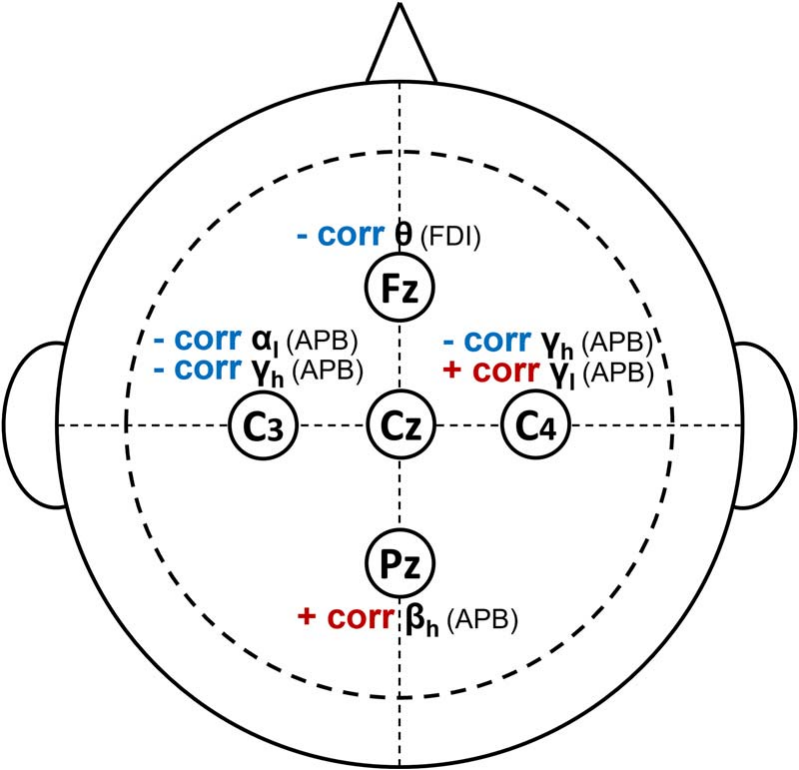
Significant correlations of the CMC with clinically defined UMN dysfunction score show location-specific positivity and negativity.

PLS participants with greater clinical impairment exhibited larger CMC in brain regions which are not directly associated with motor execution (positive correlations in Fig. 6 between APB and the ipsilateral motor cortex, C4, and the parietal region, Pz). This finding suggests that PLS affects a wider brain network extending beyond M1, as indicated in previous neuroimaging studies (Finegan et al. 2019). Less-impaired PLS participants exhibited higher alpha- and gamma-band coherence in contralateral M1. The significant correlations between CMC and UMN score were primarily observed in the APB muscle (5/6 correlations), though the reason for this is unclear. Previous studies have found no evidence that PLS conforms to the “split-hand plus” feature of ALS, whereby greater weakness and atrophy is observed in APB relative to other muscles innervated by the median nerve (Menon et al. 2013).

PLS participants with greater motor impairments exhibited higher beta-band CMC in the parietal area (Pz) (Fig. 6). Studies in nonhuman primates have shown that activity in the posterior parietal sites is modulated by beta-band oscillations from the somatosensory cortex and that they, in turn, exert an influence on the motor cortex (Brovelli et al. 2004). Though the majority of corticospinal neurons originate from M1, the neuroanatomical and electrophysiological studies in primates have also found evidence of corticospinal projections from the supplementary motor area and somatosensory and parietal cortices (Murray and Coulter 1981; Galea and Darian-Smith 1994; Maier et al. 2002). CMC at EEG electrodes over non-M1 cortical areas could thus occur due to an increase in the relative contribution of alternative descending pathways to muscle activation, other than direct M1 projections. These synchronies could also reflect a restructuring of cortico-cortical communication between non-M1 regions and areas such as M1 that have direct projections to the spinal motor pools. For example, the enhanced beta-band coupling between the parietal brain region and muscular activity could reflect an increase in the functional connectivity of these brain networks (Meoded et al. 2015). It is also possible that the chronic loss of corticospinal input to the spinal motoneurons, which is combined with extreme muscle weakness and slowing of movement in PLS, could produce a change in afferent activity. This would in turn influence CMC. Though beta-band CMC is primarily driven by efferent supraspinal structures, there is now evidence to suggest that it can be modulated by sensory receptors that provide afferent feedback to the central nervous system (Witham et al. 2011).

Although the observed CMC differences in PLS could arise from both the direct and indirect effects of UMN degeneration, the increased CMC in more-impaired PLS participants for specific brain regions could potentially suggest that these changes are compensatory/adaptive in nature. Taken together, these results could suggest that the pattern of brain network reorganization in PLS follows a similar trajectory to recovery in stroke, where more-impaired PLS participants rely on contributions from the ipsilateral hemisphere, but those who are minimally affected can recover function by restructuring the functional connectivity in the contralateral hemisphere (Brancaccio et al. 2022). Future studies are needed to elucidate the pathways through which these wider brain regions could influence muscle activity and determine the exact nature of the observed changes in CMC (pathogenic, adaptive, or compensatory). These network-level changes could be further characterized in the future longitudinal studies of PLS by examining the changes in the CMC measures alongside changes in the clinical scores of UMN impairment.

### Future directions

The novel findings of this study identify distinct differences in the CMC patterns found in PLS. Though the data presented in the current study cannot determine the exact mechanism and/or neuro-anatomical pathways through which cortical signals originating outside of contralateral M1 influence muscle activity, we suggest several possible mechanisms through which abnormal CMC in PLS could arise.

Future studies could also examine whether these CMC patterns can discriminate PLS from more rapidly progressive ALS phenotypes. There is a clear need for quantitative measures to support diagnosis, as people with PLS currently have long periods of diagnostic uncertainty and face exclusion from ALS clinical trials (people with restricted UMN symptoms and suspected PLS typically do not meet inclusion criteria) (Brooks et al. 2000; D'Amico et al. 2013). The differences between more- and less-impaired PLS participants further suggest that CMC has the potential for development as a tool to monitor disease progression or importantly as a measure to assess target engagement in clinical trials (Jeromin and Bowser 2017). These measures are particularly needed for PLS, as longitudinal progression is difficult to quantify in such a slowly progressing disease. The PLS-specific differences in CMC and the differences between more- and less-impaired PLS participants reported in this study provide the basis for further development of these markers of motor network dysfunction.

## Conclusion

This study demonstrates the presence of abnormal corticomuscular coherence in PLS for the first time, which we suggest could reflect a restructuring of the cortical network connectivity in response to UMN degeneration. This observation suggests that PLS affects a sensorimotor brain network extending beyond the primary motor cortex. Correlations showed that higher CMC in specific brain regions was also observed in more-impaired PLS participants compared with those with less severe impairments. This may suggest these differences are compensatory/adaptive in nature, though these differences could arise from both the direct and indirect effects of UMN degeneration. The correlations with clinical UMN scores demonstrate the potential for CMC measurements to be used as a tool to identify dysfunction in specific cortical networks during motor tasks and prompt further development of quantitative neurophysiology-based biomarker candidates in PLS.

## Supplementary Material

Supplementary_Material_S1_bhad152

Supplementary_Material_S2_bhad152

Supplementary_Material_S3_bhad152

Suppelmentary_Material_S4_bhad152

Supplementary_Material_S5_bhad152

Supplementary_Material_S6_bhad152

Supplementary_Material_bhad152

## Data Availability

The data that support the findings of this study are available from the corresponding author upon reasonable request and subject to the approvals by Data Protection Officer and Technology Transfer Office in Trinity College Dublin.
