# Supplementary material for: Cortico-muscular coherence in primary lateral sclerosis reveals abnormal cortical engagement during motor function beyond primary motor areas
Source: Cereb Cortex. 2023 May 4;33(13):8712–23. doi: 10.1093/cercor/bhad152 (PMC10321081; doi:10.1093/cercor/bhad152)
Supplement: Supplementary_Material_S1_bhad152 [file supplementary_material_s1_bhad152.docx]

***Verification of the task-effect: CMC levels in two motor tasks in healthy participants***

In our pilot experiments in the control group, the pincer grip task generated low levels of beta-band CMC when compared with precision grip task (Coffey et al. 2020), suggesting that this task may be more suitable for studying abnormally increased CMC patterns. The results of the pilot experiments are shown in Figure S1, depicting both classical and banded group average CMC in controls for the 10% MVC pincer grip task and the precision grip task. During precision grip task, the controls showed clear and significant beta CMC peaks at group level.

The 10% MVC pincer grip task exhibited a lower CMC peak in the beta-band when compared with CMC during precision grip, S1. However, significant beta-band coherence was still detected during the pincer grip task in 14 out of 18 control subjects. The lower CMC observed in the pincer grip task is expected as the force is exerted against a rigid load cell with no digit displacement and no object flexibility. Previous studies have shown that beta-band CMC is lower for isometric pinch grip contractions against a rigid force transducer when compared with those performed with a compliant, or spring-like load (Kilner et al. 2000).


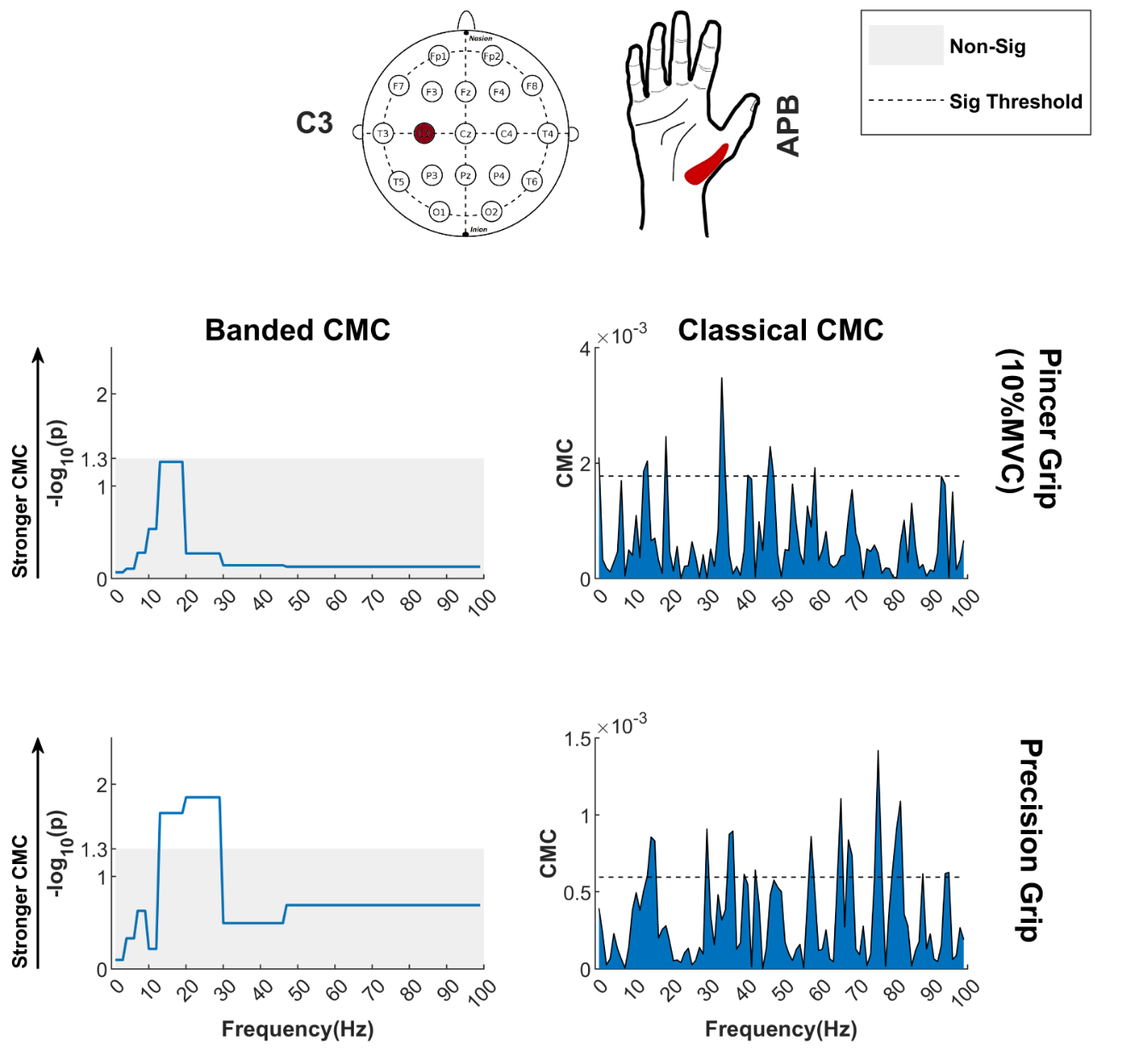


**Figure S1. Classical and banded group average Corticomuscular coherence (CMC) for healthy controls between C3 (contralateral primary motor cortex) and Abductor Pollicis Brevis (APB) muscle during pincer grip at 10% maximum voluntary contraction (top panel) and precision grip (bottom panel) using thumb and index finger of right hand.** The comparison confirms that the pincer grip task generates lower level of typical beta CMC compared to precision grip task (this task was chosen as it was hypothesised that a lower level of beta CMC would facilitate the detection of abnormally-increased CMC in the PLS group).

**References**

Coffey A, Bista S, Fasano A, Buxo T, Mitchell M, Giglia ER, Dukic S, Fenech M, Barry M, Wade A, Heverin M, Muthuraman M, Carson RG, Lowery M, Hardiman O, Nasseroleslami B. 2020. Altered supraspinal motor networks in survivors of poliomyelitis: A cortico-muscular coherence study. Clin Neurophysiol. 132:106-113.

Kilner JM, Baker SN, Salenius S, Hari R, Lemon RN. 2000. Human Cortical Muscle Coherence Is Directly Related to Specific Motor Parameters. The Journal of Neuroscience. 20:8838-8845.
