# Supplementary material for: Cortico-muscular coherence in primary lateral sclerosis reveals abnormal cortical engagement during motor function beyond primary motor areas
Source: Cereb Cortex. 2023 May 4;33(13):8712–23. doi: 10.1093/cercor/bhad152 (PMC10321081; doi:10.1093/cercor/bhad152)
Supplement: Supplementary_Material_S2_bhad152 [file supplementary_material_s2_bhad152.docx]

***CMC in healthy participants***


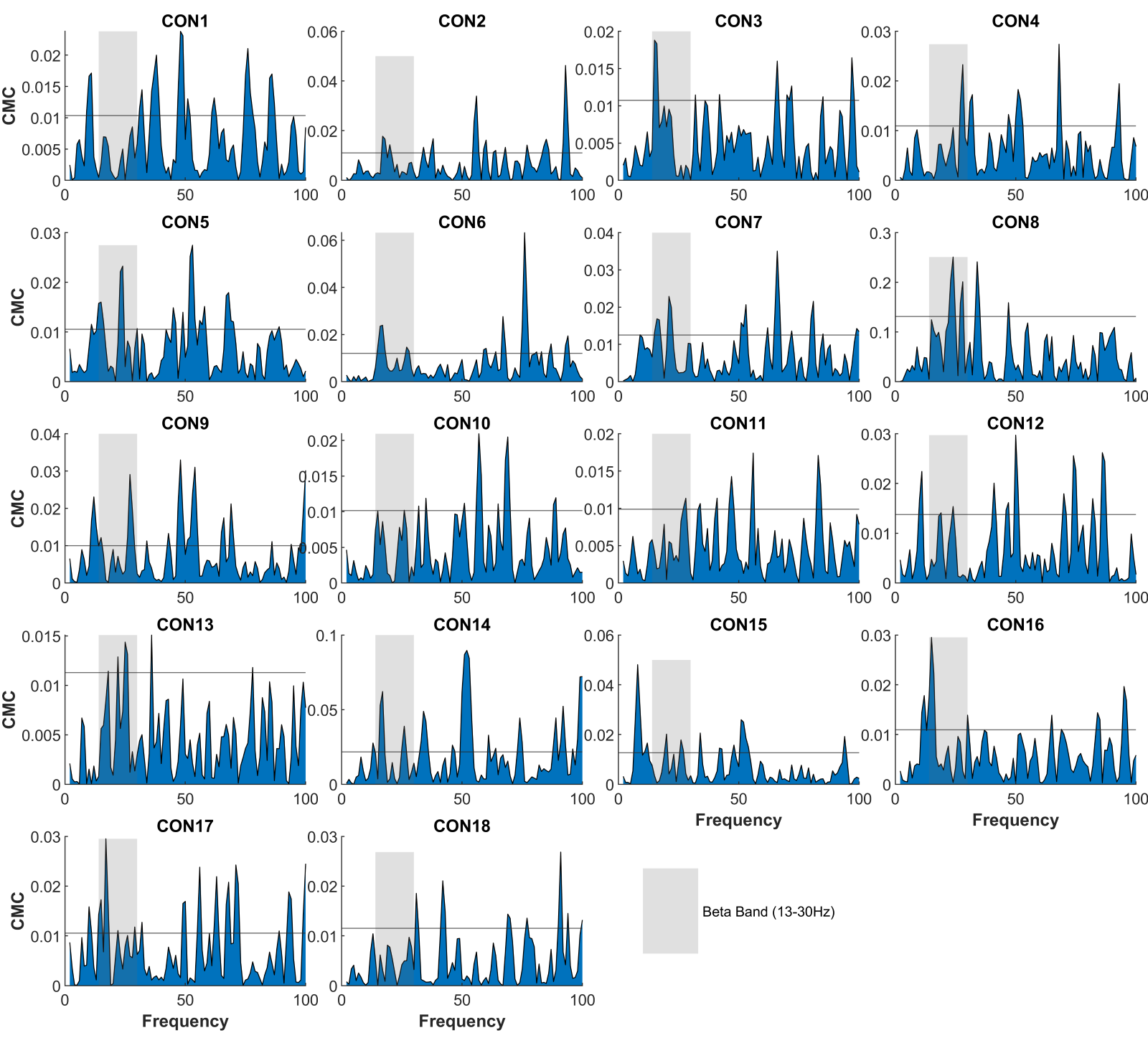


**Figure S2.** Individual classical magnitude squared CMC between C3 (contralateral primary motor cortex) and Abductor Pollicis Brevis (APB) muscle for pincer grip (10% MVC) task. The significant threshold or estimate of upper 95% confidence limit for classical CMC is calculated as $1- {0.05}^{\frac{1}{\left( L-1 \right)*0.375}}$ , where L is the number of trials used to calculate coherence.
