# Supplementary material for: Cortico-muscular coherence in primary lateral sclerosis reveals abnormal cortical engagement during motor function beyond primary motor areas
Source: Cereb Cortex. 2023 May 4;33(13):8712–23. doi: 10.1093/cercor/bhad152 (PMC10321081; doi:10.1093/cercor/bhad152)
Supplement: Supplementary_Material_S3_bhad152 [file supplementary_material_s3_bhad152.docx]

See the Methods section “Estimation of Coherence Spectrum and Banded Coherence” for a description of the procedure for calculating the banded coherence and the classical (or magnitude-squared) coherence.

***Group average banded Corticomuscular coherence (CMC) for all EEG and EMG channels***


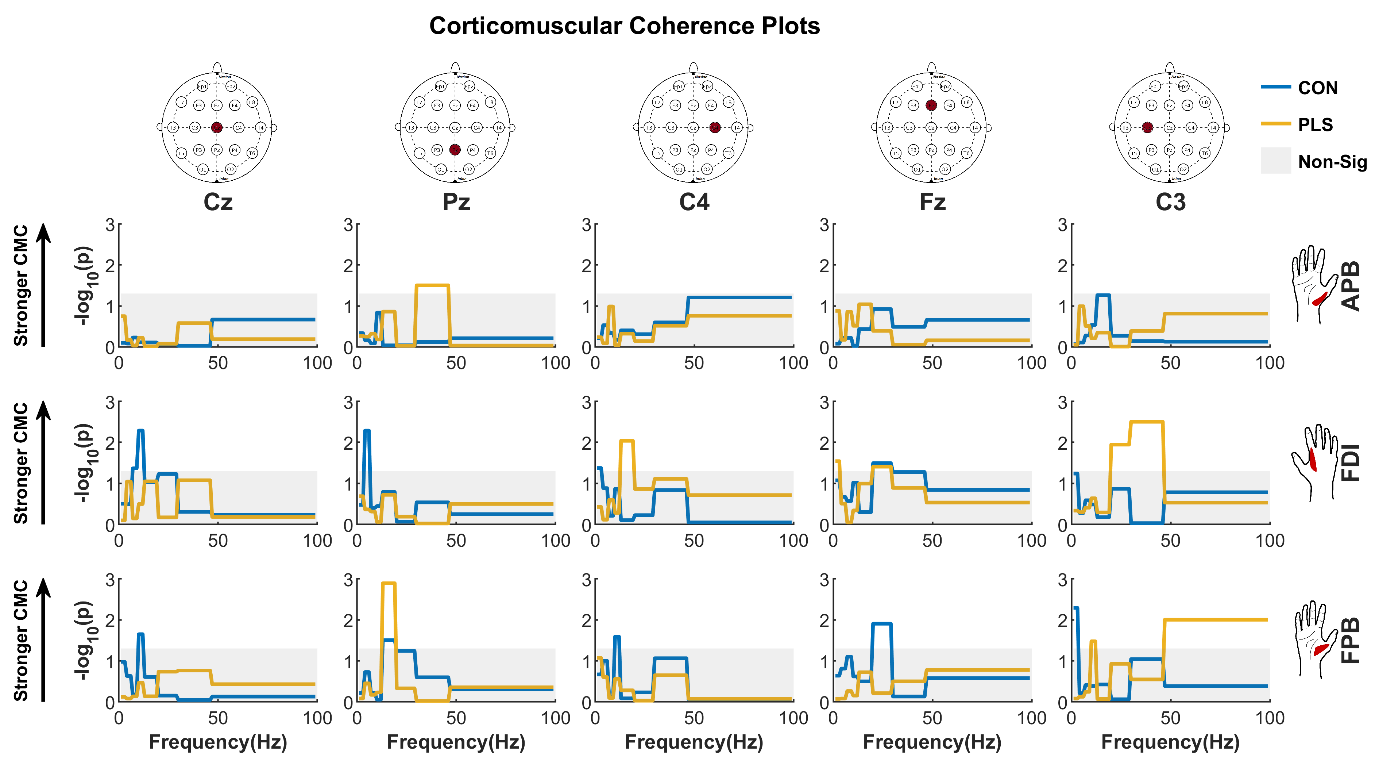


**Figure S3.** Group average banded cortico-muscular coherence (CMC) across 5 selected EEG and 3 selected EMG channels in the PLS cohort vs. Healthy Controls. The EEG channels (C3, Cz, C4, Pz, and Fz) are surface Laplacian-referenced and the EMG channels are bipolar surface EMG channels. The CMC were corrected for multiple comparison using adaptive FDR at q = 0.05. The coherence spectra were grouped over pre-defined bands using the spatial median (“pCoh”). The CMC values that were significantly different between PLS and control groups are outlined in Figure 3 in the main manuscript.
