# Supplementary material for: Cortico-muscular coherence in primary lateral sclerosis reveals abnormal cortical engagement during motor function beyond primary motor areas
Source: Cereb Cortex. 2023 May 4;33(13):8712–23. doi: 10.1093/cercor/bhad152 (PMC10321081; doi:10.1093/cercor/bhad152)
Supplement: Suppelmentary_Material_S4_bhad152 [file suppelmentary_material_s4_bhad152.docx]

See the Methods section “Estimation of Coherence Spectrum and Banded Coherence” for a description of the procedure for calculating the banded coherence and the classical (or magnitude-squared) coherence.

***Group average classical Corticomuscular coherence (CMC) for all EEG and EMG channels***

Note that the group average of Classical CMC spectra (Figure S4) is similar to the group average of banded CMC spectra (Stouffer’s averaging of p values-based CMC) (Figure S3) for both PLS cohort and Healthy Controls.


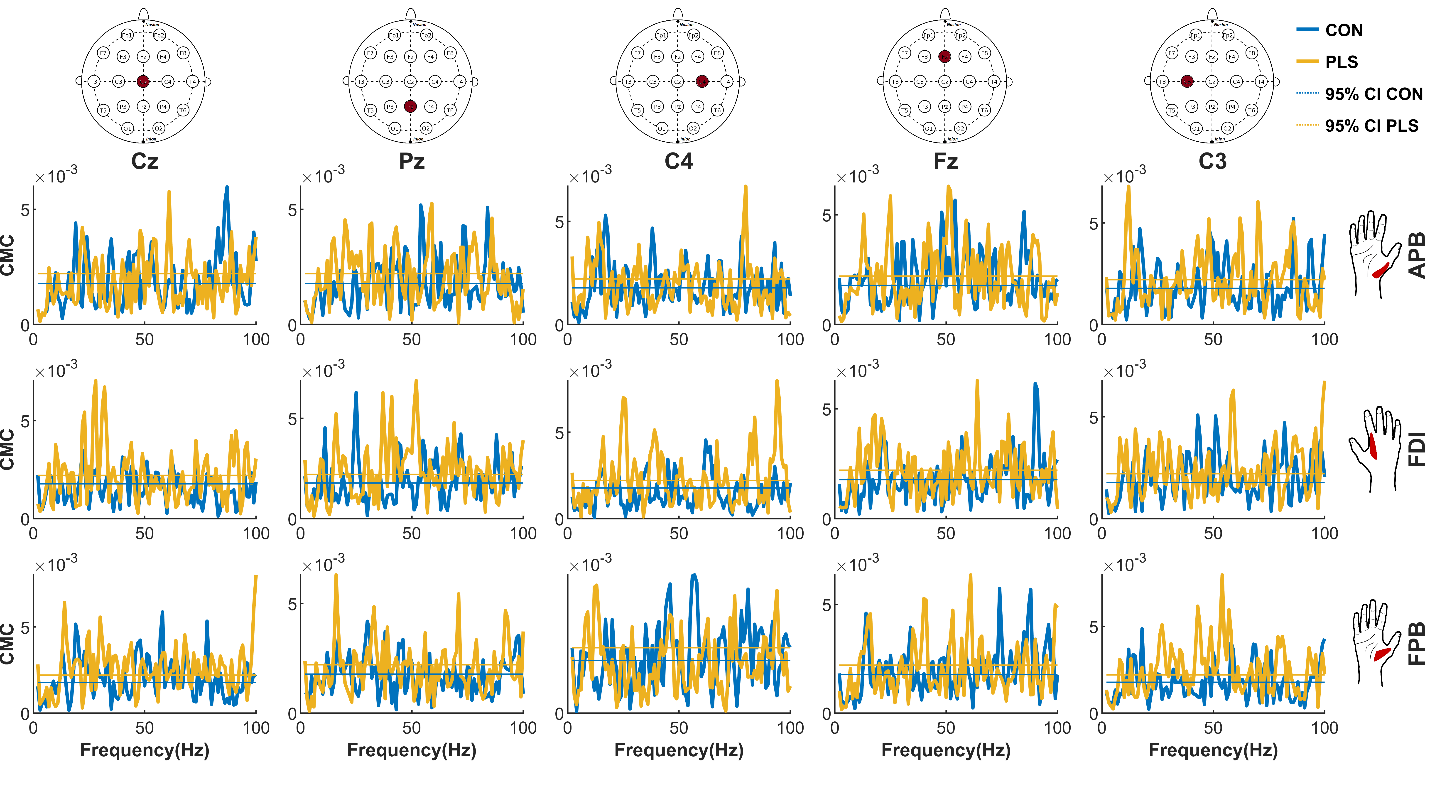
**Figure S4.** Group average classical magnitude-squared CMC across 5 selected EEG and 3 selected EMG channels in the PLS cohort vs. Healthy Controls. The EEG channels (C3, Cz, C4, Pz, and Fz) are surface Laplacian-referenced and the EMG channels are bipolar surface EMG channels.
