# Supplementary material for: Cortico-muscular coherence in primary lateral sclerosis reveals abnormal cortical engagement during motor function beyond primary motor areas
Source: Cereb Cortex. 2023 May 4;33(13):8712–23. doi: 10.1093/cercor/bhad152 (PMC10321081; doi:10.1093/cercor/bhad152)
Supplement: Supplementary_Material_S5_bhad152 [file supplementary_material_s5_bhad152.docx]

***Spatial Topology of beta CMC for Controls***

The spatial topology of banded beta “pCoh” CMC between EMG and the five EEG channels showed maximum CMC within the sensorimotor cortices (C3, Cz) and visuomotor processing areas (Pz) in controls (Fig. S5 top panel). Similar results were observed with classical magnitude-squared CMC (Fig. S5 bottom panel), however the banded pCoh resulted in more localised CMC patterns.


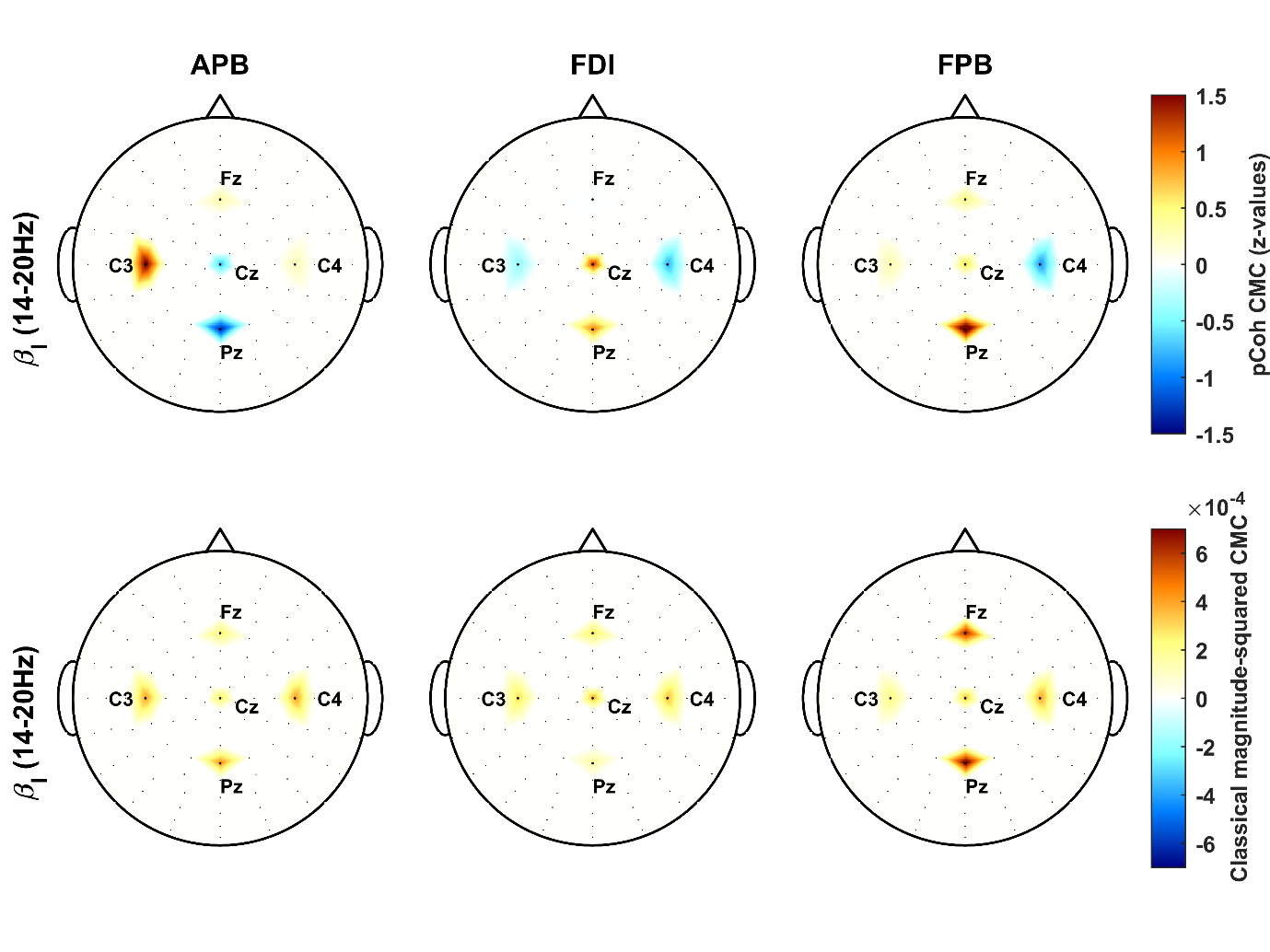


**Figure S5.** The spatial topology of group average beta CMC between 5 EEG (C3, C4, Cz, Fz, Pz) and 3 EMG (APB, FDI, FPB) channels using banded “pCoh” CMC method (top panel) and classical magnitude-squared CMC method in the same band (bottom panel) in healthy controls.
