# Supplementary material for: Cortico-muscular coherence in primary lateral sclerosis reveals abnormal cortical engagement during motor function beyond primary motor areas
Source: Cereb Cortex. 2023 May 4;33(13):8712–23. doi: 10.1093/cercor/bhad152 (PMC10321081; doi:10.1093/cercor/bhad152)
Supplement: Supplementary_Material_S6_bhad152 [file supplementary_material_s6_bhad152.docx]

***Comparing CMC in the PLS cohort using different spectral averaging and banded coherence***


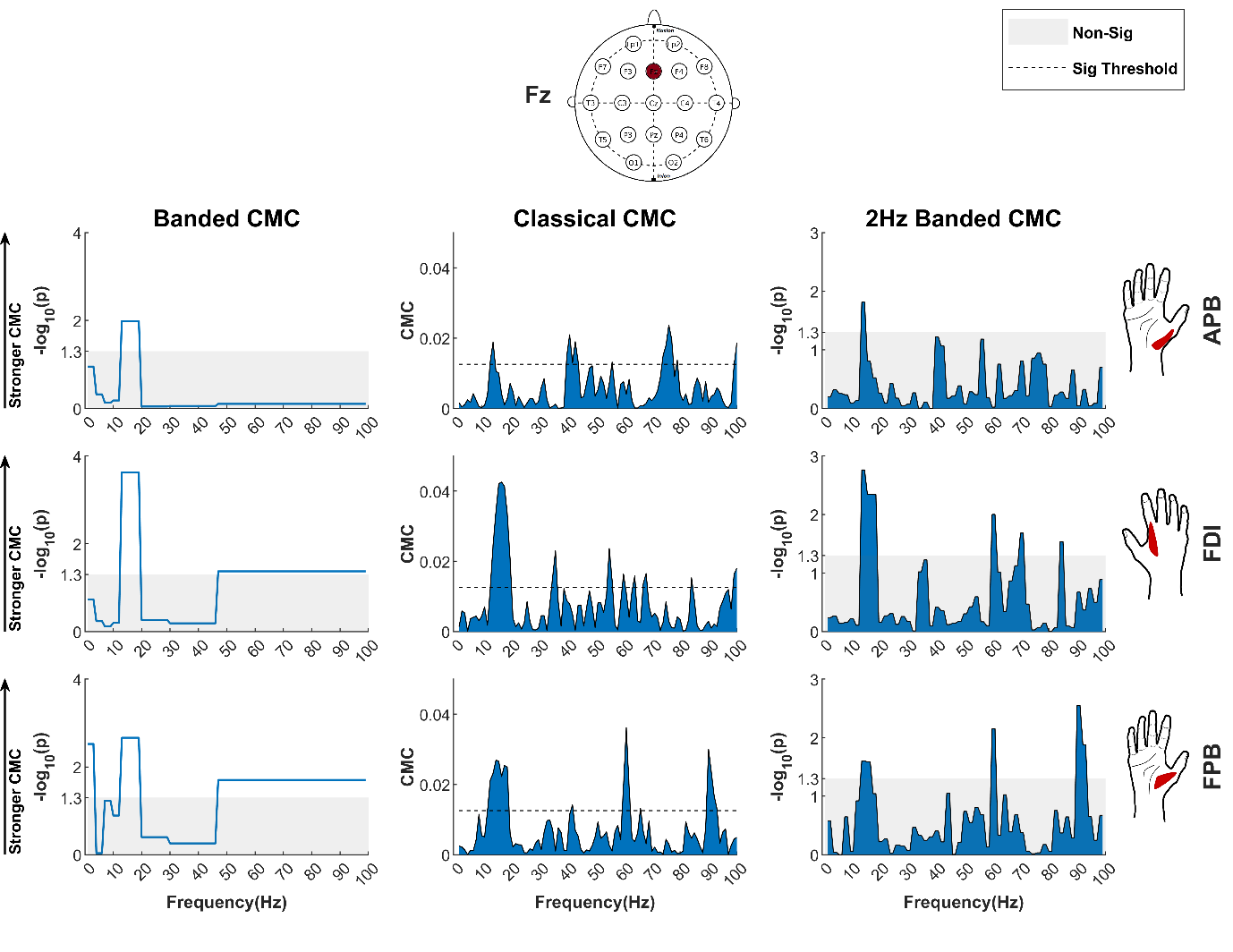


**Figure S6.** Banded and classical magnitude-squared CMC between Fz and 3 EMG channels in a PLS participant. Note that both methods have detected significant CMC in low-beta (14-20 Hz) and high-gamma (53-97Hz) bands except that the significant CMC detected by the banded method (“pCoh” with 2 Hz band intervals) is more pronounced. The significance threshold or estimate of upper 95% confidence limit for classical CMC is calculated as $1- {0.05}^{\frac{1}{(L-1)*0.375}}$ , where L is the number of trials used to calculate coherence.
