# Supplementary material for: Cortico-muscular coherence in primary lateral sclerosis reveals abnormal cortical engagement during motor function beyond primary motor areas
Source: Cereb Cortex. 2023 May 4;33(13):8712–23. doi: 10.1093/cercor/bhad152 (PMC10321081; doi:10.1093/cercor/bhad152)
Supplement: Supplementary_Material_bhad152 [file supplementary_material_bhad152.docx]

**Supplementary Materials**

**Section 1: Comparison of banded and classical CMC**

See the Methods section “Estimation of Coherence Spectrum and Banded Coherence” for a description of the procedure for calculating the banded coherence and the classical (or magnitude-squared) coherence.

***1.1 Group average banded Corticomuscular coherence (CMC) for all EEG and EMG channels***


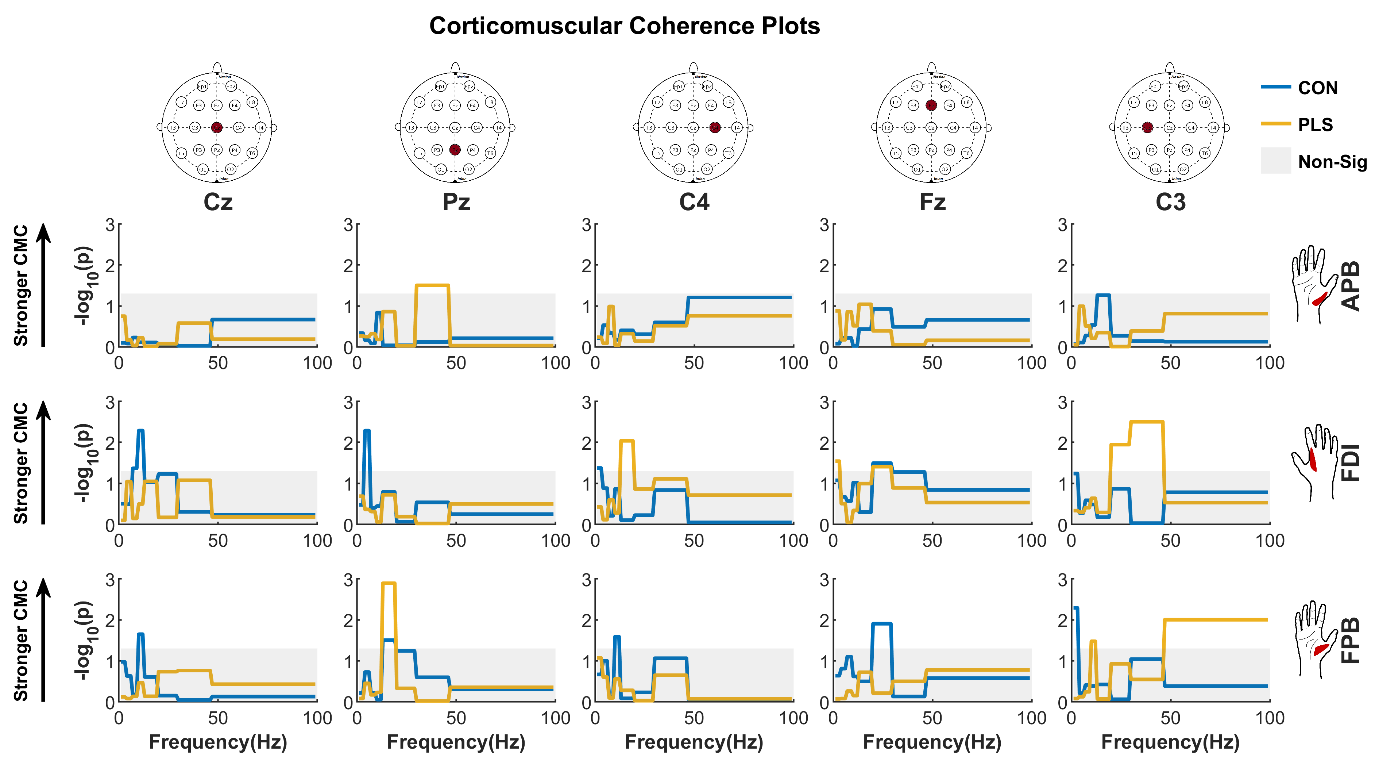


**Figure S1.** Group average banded cortico-muscular coherence (CMC) across 5 selected EEG and 3 selected EMG channels in the PLS cohort vs. Healthy Controls. The EEG channels (C3, Cz, C4, Pz, and Fz) are surface Laplacian-referenced and the EMG channels are bipolar surface EMG channels. The CMC were corrected for multiple comparison using adaptive FDR at q = 0.05. The coherence spectra were grouped over pre-defined bands using the spatial median (“pCoh”). The CMC values that were significantly different between PLS and control groups are outlined in Figure 3 in the main manuscript.

***1.2 Group average classical Corticomuscular coherence (CMC) for all EEG and EMG channels***

Note that the group average of Classical CMC spectra (Figure S2) is similar to the group average of banded CMC spectra (Stouffer’s averaging of p values-based CMC) (Figure S1) for both PLS cohort and Healthy Controls.


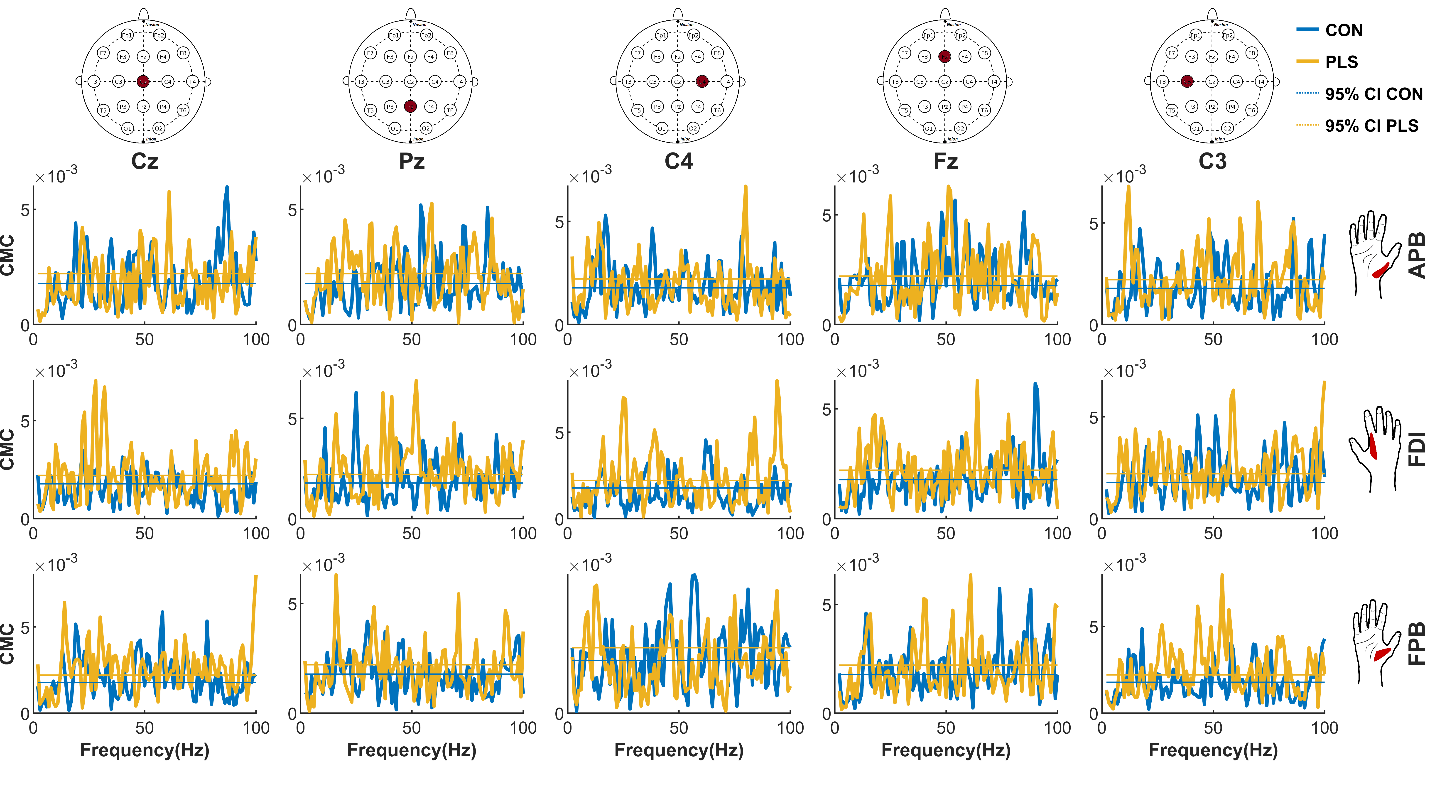
**Figure S2.** Group average classical magnitude-squared CMC across 5 selected EEG and 3 selected EMG channels in the PLS cohort vs. Healthy Controls. The EEG channels (C3, Cz, C4, Pz, and Fz) are surface Laplacian-referenced and the EMG channels are bipolar surface EMG channels.

***1.3 Comparing CMC in the PLS cohort using different spectral averaging and banded coherence***


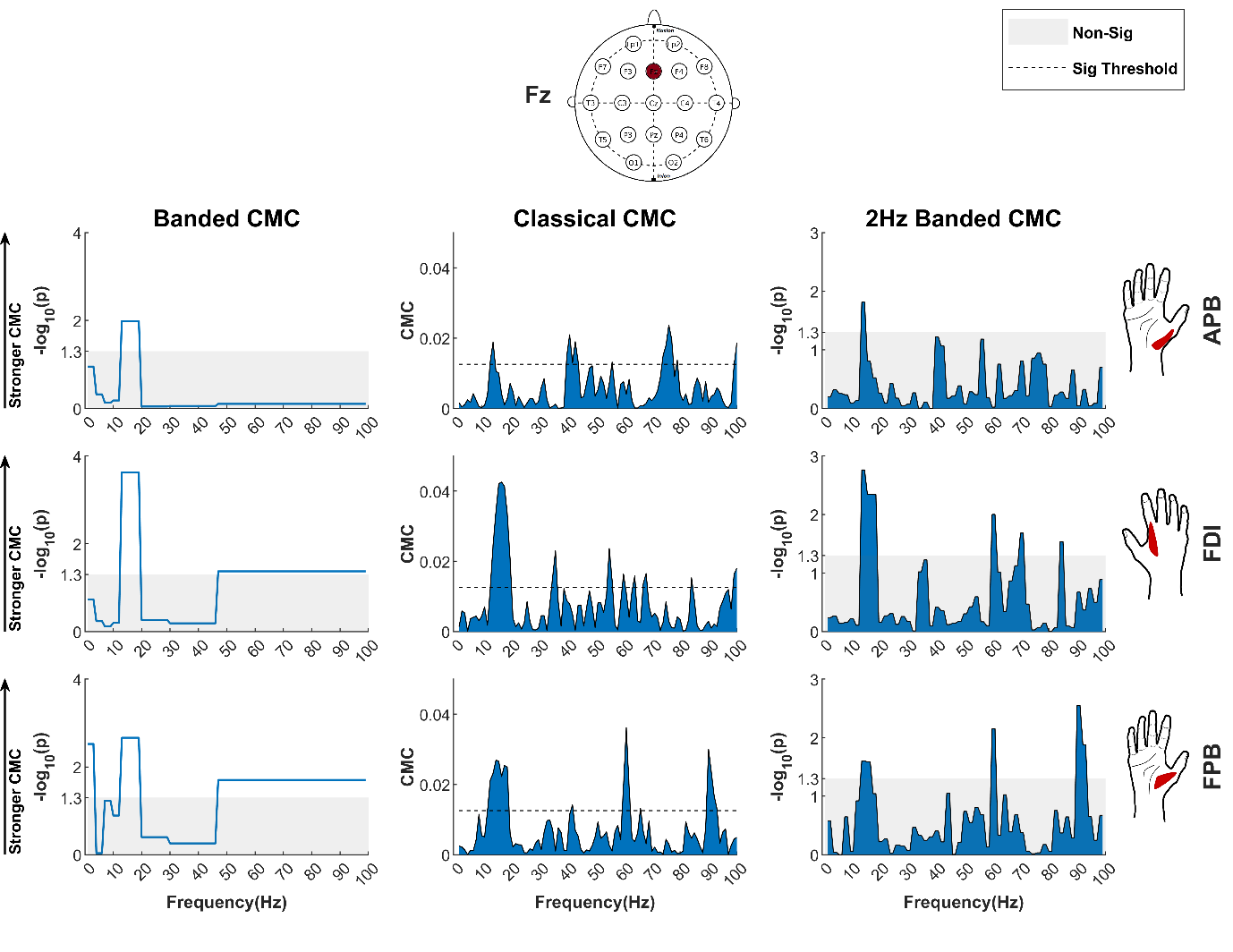


**Figure S3.** Banded and classical magnitude-squared CMC between Fz and 3 EMG channels in a PLS participant. Note that both methods have detected significant CMC in low-beta (14-20 Hz) and high-gamma (53-97Hz) bands except that the significant CMC detected by the banded method (“pCoh” with 2 Hz band intervals) is more pronounced. The significance threshold or estimate of upper 95% confidence limit for classical CMC is calculated as $1- {0.05}^{\frac{1}{(L-1)*0.375}}$ , where L is the number of trials used to calculate coherence.

***1.4 Spatial Topology of beta CMC for Controls***

The spatial topology of banded beta “pCoh” CMC between EMG and the five EEG channels showed maximum CMC within the sensorimotor cortices (C3, Cz) and visuomotor processing areas (Pz) in controls (Fig. S4 top panel). Similar results were observed with classical magnitude-squared CMC (Fig. S4 bottom panel), however the banded pCoh resulted in more localised CMC patterns.


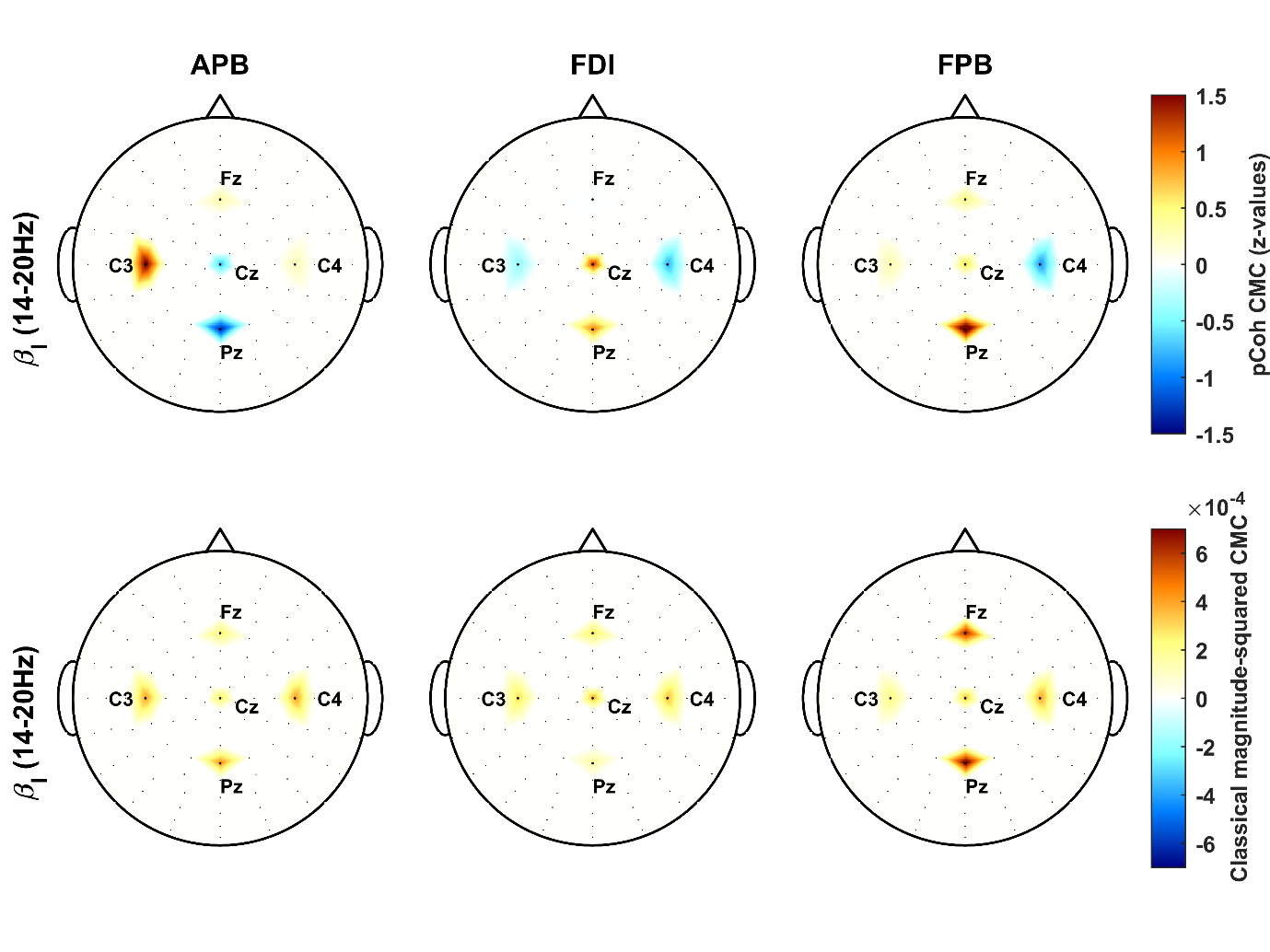


**Figure S4.** The spatial topology of group average beta CMC between 5 EEG (C3, C4, Cz, Fz, Pz) and 3 EMG (APB, FDI, FPB) channels using banded “pCoh” CMC method (top panel) and classical magnitude-squared CMC method in the same band (bottom panel) in healthy controls.

**Section 2: Choice of task**

***2.1 Verification of the task-effect: CMC levels in two motor tasks in healthy participants***

In our pilot experiments in the control group, the pincer grip task generated low levels of beta-band CMC when compared with precision grip task (Coffey et al. 2020), suggesting that this task may be more suitable for studying abnormally increased CMC patterns. The results of the pilot experiments are shown in Figure S5, depicting both classical and banded group average CMC in controls for the 10% MVC pincer grip task and the precision grip task. During precision grip task, the controls showed clear and significant beta CMC peaks at group level.

The 10% MVC pincer grip task exhibited a lower CMC peak in the beta-band when compared with CMC during precision grip, S5. However, significant beta-band coherence was still detected during the pincer grip task in 14 out of 18 control subjects. The lower CMC observed in the pincer grip task is expected as the force is exerted against a rigid load cell with no digit displacement and no object flexibility. Previous studies have shown that beta-band CMC is lower for isometric pinch grip contractions against a rigid force transducer when compared with those performed with a compliant, or spring-like load (Kilner et al. 2000).


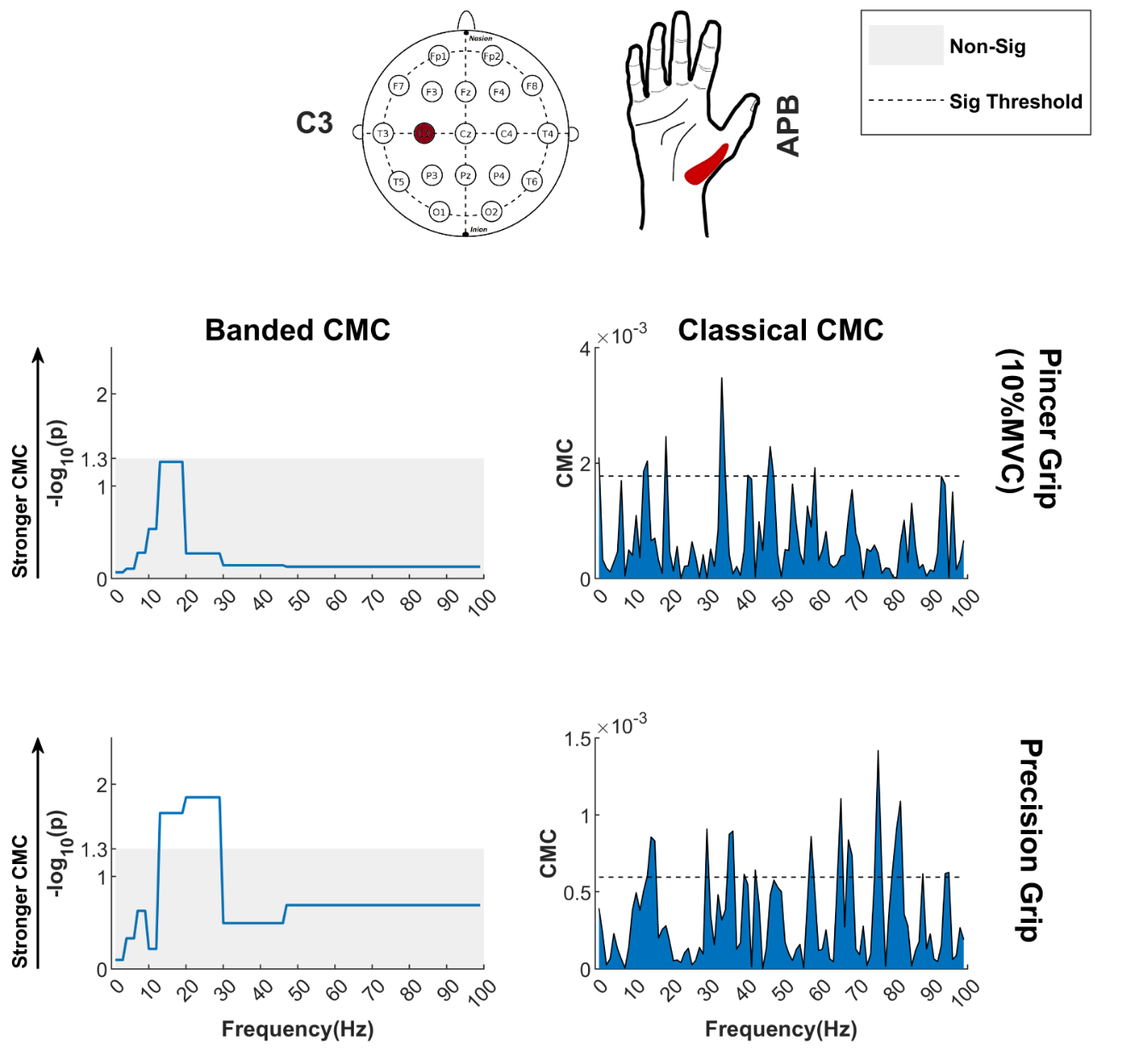


**Figure S5. Classical and banded group average Corticomuscular coherence (CMC) for healthy controls between C3 (contralateral primary motor cortex) and Abductor Pollicis Brevis (APB) muscle during pincer grip at 10% maximum voluntary contraction (top panel) and precision grip (bottom panel) using thumb and index finger of right hand.** The comparison confirms that the pincer grip task generates lower level of typical beta CMC compared to precision grip task (this task was chosen as it was hypothesised that a lower level of beta CMC would facilitate the detection of abnormally-increased CMC in the PLS group).

***2.2 CMC in healthy participants***


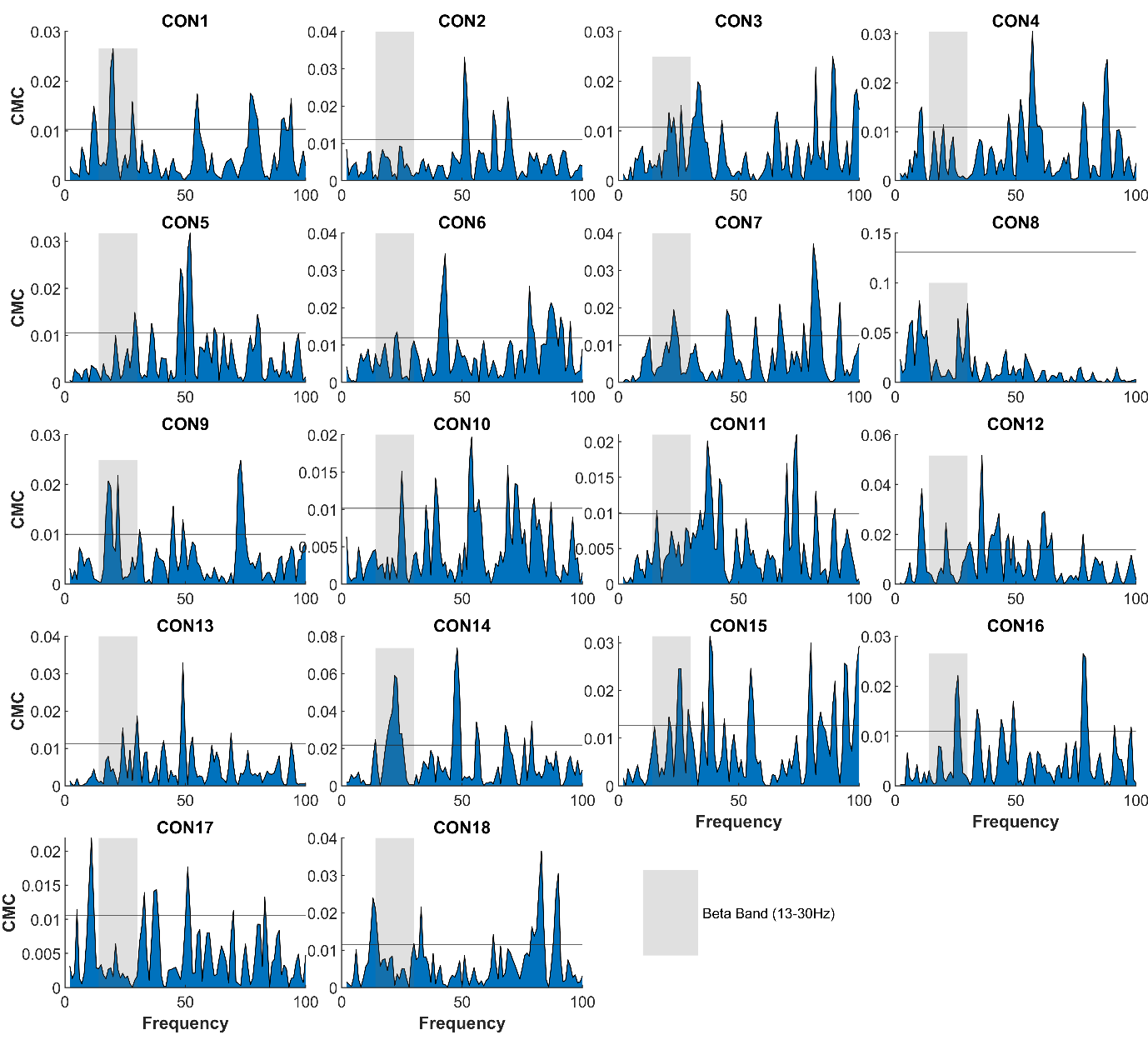
**Figure S6.** Individual classical magnitude squared CMC between C3 (contralateral primary motor cortex) and Flexor Pollicis Brevis (FDI) muscle for pincer grip (10% MVC) task. The significant threshold or estimate of upper 95% confidence limit for classical CMC is calculated as $1- {0.05}^{\frac{1}{\left( L-1 \right)*0.375}}$ , where L is the number of trials used to calculate coherence.

**References**

Coffey A, Bista S, Fasano A, Buxo T, Mitchell M, Giglia ER, Dukic S, Fenech M, Barry M, Wade A, Heverin M, Muthuraman M, Carson RG, Lowery M, Hardiman O, Nasseroleslami B. 2020. Altered supraspinal motor networks in survivors of poliomyelitis: A cortico-muscular coherence study. Clin Neurophysiol. 132:106-113.

Kilner JM, Baker SN, Salenius S, Hari R, Lemon RN. 2000. Human Cortical Muscle Coherence Is Directly Related to Specific Motor Parameters. The Journal of Neuroscience. 20:8838-8845.
